# Stress‐induced activation of the proline biosynthetic pathway in *Bacillus subtilis*: a population‐wide and single‐cell study of the osmotically controlled *proHJ* promoter

**DOI:** 10.1111/1751-7915.14073

**Published:** 2022-05-20

**Authors:** Luiza P. Morawska, Ruud G. J. Detert Oude Weme, Elrike Frenzel, Maarten Dirkzwager, Tamara Hoffmann, Erhard Bremer, Oscar P. Kuipers

**Affiliations:** ^1^ 3647 Molecular Genetics Group Groningen Biomolecular Sciences and Biotechnology Institute University of Groningen Nijenborgh 7 9747 AG Groningen The Netherlands; ^2^ Department of Biology Laboratory for Microbiology Philipps University Marburg Karl‐von‐Frisch‐Str.8 D‐35032 Marburg Germany; ^3^ Center for Synthetic Microbiology (SYNMIKRO) Philipps‐University Marburg Karl‐von‐Frisch Strasse 14 35043 Marburg Germany

## Abstract

*Bacillus subtilis*, in its natural habitat, is regularly exposed to rapid changes in the osmolarity of its surrounding. As its primary survival strategy, it accumulates large amounts of the compatible solute proline by activating the de novo proline biosynthesis pathway and exploiting the glutamate pools. This osmotically‐induced biosynthesis requires activation of a SigA‐type promoter that drives the expression of the *proHJ* operon. Population‐wide studies have shown that the activity of the *proHJ* promoter correlates with the increased osmotic pressure of the environment. Therefore, the activation of the *proHJ* transcription should be an adequate measure of the adaptation to osmotic stress through proline synthesis in the absence of other osmoprotectants. In this study, we investigate the kinetics of the *proHJ* promoter activation and the early adaptation to mild osmotic upshift at the single‐cell level. Under these conditions, we observed a switching point and heterogeneous proline biosynthesis gene expression, where the subpopulation of cells showing active *proHJ* transcription is able to continuously divide, and those unresponsive to osmotic stress remain dormant. Additionally, we demonstrate that bactericidal antibiotics significantly upregulate *proHJ* transcription in the absence of externally imposed osmotic pressure, suggesting that the osmotically‐controlled proline biosynthesis pathway is also involved in the antibiotic‐mediated stress response.

## Graphical abstract

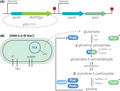

## Introduction

**Fig. 1:**
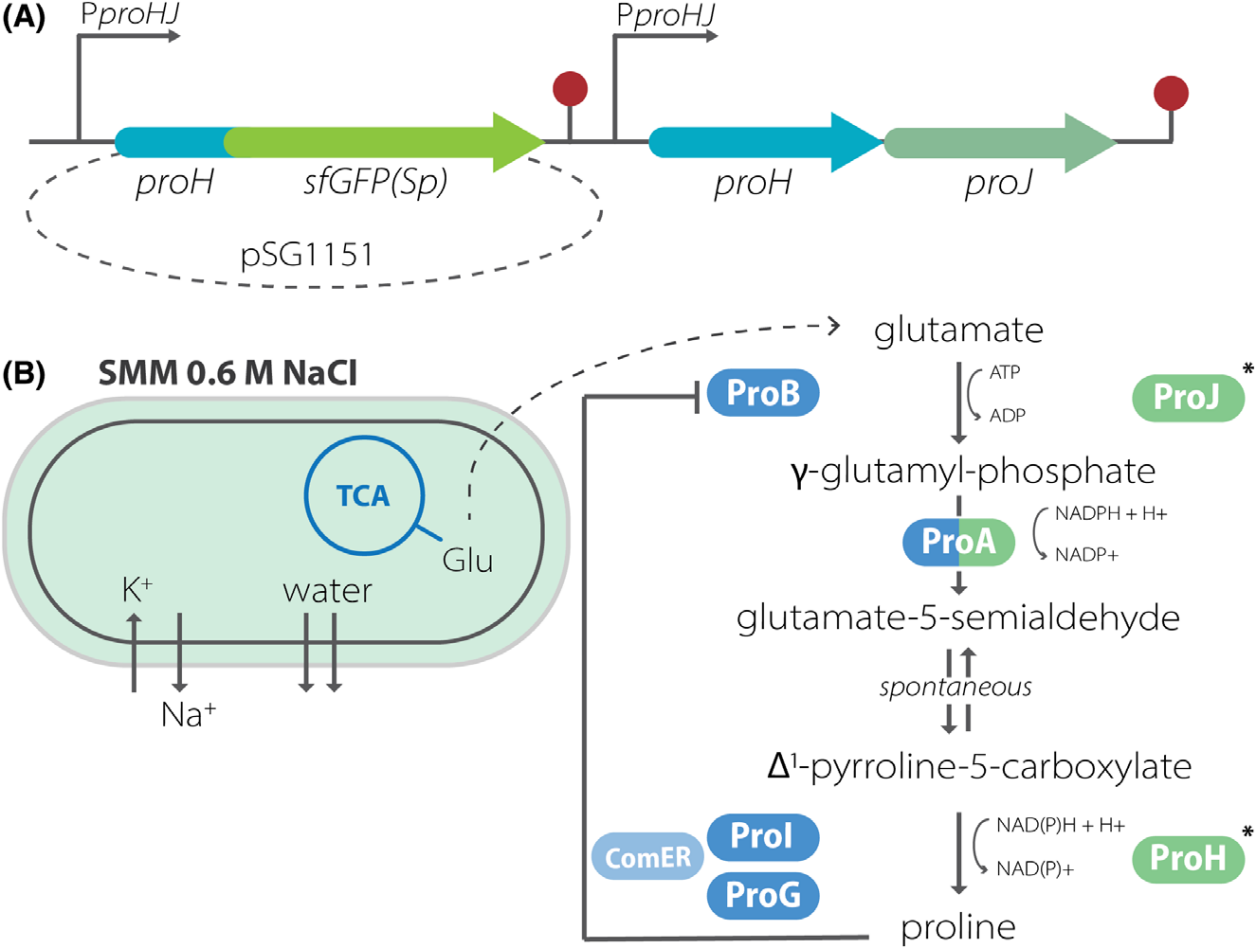
(A) Schematic representation of the insertion of the reporter construct into a genomic locus in *B. subtilis* 168. The pSG1151 plasmid containing homologous *proH* sequence fused with *sfGFP (Sp)* was integrated into the chromosome in a Campbell‐like manner, upstream the native *proHJ* operon. (B) *B. subtilis* 168 response to mild osmostress (0.6 M NaCl) in SMM media. Enzymatic reactions related to osmotically induced proline biosynthesis are coloured in green, whereas the non‐stress related, anabolic pathway is marked with blue. Asterisk indicates products of *proHJ* translation followed in this study.

Microorganisms in their natural habitat are constantly challenged with rapid changes of their surroundings. For instance, soil‐inhabiting bacteria frequently encounter interchanging periods of flooding and drying of the soil's topmost layers, causing osmotic stress (Hoffmann and Bremer, 2016). When the osmolarity of the environment fluctuates, the integrity of the semi‐permeable cell membrane and sufficient hydration of the cytoplasm are challenged with passive water fluxes in and out of the cell (Bremer and Krämer, 2019). Therefore, bacteria employ various adaptive mechanisms to maintain cell turgor and minimize cellular damage. As an initial response to high osmolarity conditions, microbes must counteract the outflow of intracellular water by amassing large quantities of potassium and accumulating highly soluble organic osmolytes, known as compatible solutes (Csonka, 1989; Whatmore *et al*., 1990; Wood *et al*., 2001; Godard *et al*., 2020). Importantly, unlike most ions, compatible solutes do not interfere with cellular functions because of their physicochemical properties. These physiologically compliant organic osmolytes can accumulate to high levels, maintain the cell's turgor and avoid over‐crowding of the cytoplasm even in high osmolarity environments (Csonka, 1989; Kempf and Bremer, 1998; van den Berg *et al*., 2017). In *Bacillus subtilis*, a Gram‐positive bacterium typically found in the upper layers of the soil (Earl *et al*., 2008), the initial uptake of potassium ions is followed by accumulation and/or synthesis of proline (from glutamate) and glycine betaine (from the acquired choline pools) (Kempf and Bremer, 1998; Brill *et al*., 2011a; Hoffmann *et al*., 2012; Zaprasis *et al*., 2015). However, in contrast to all other osmoprotectants exploited by *B. subtilis*, proline is the only one that can be synthesized *de novo* (Whatmore *et al*., 1990; Brill *et al*., 2011a; Hoffmann *et al*., 2013). Accumulation of large amounts of proline requires the cell to re‐route its metabolism to provide precursors for its synthesis (Kohlstedt *et al*., 2014; Godard *et al*., 2020). Members of *Bacillus sp*. convert the precursor glutamate to proline in three enzymatic steps carried out sequentially by γ‐glutamyl kinase (ProB/ProJ), γ‐glutamyl phosphate reductase (ProA/ProAA) and ∆^1^‐pyrroline‐5‐carboxylate reductase (ProI/ProH/ProG/ComER) (Fig. 1B) (Belitsky *et al*., 2001; Schroeter *et al*., 2013; Forlani *et al*., 2017; Godard *et al*., 2020). Depending on the cellular demand for proline, *B. subtilis* utilizes two distinct biosynthetic pathways interlinked via the unique ProA to synthesize appropriate amounts of proline (Belitsky *et al*., 2001). Under non‐stress conditions, the cell profits from the anabolic route and activates ProB‐ProA‐ProI enzymes to support minimal proline levels sustaining protein synthesis (10 mM–20 mM) (Whatmore *et al*., 1990; Hoffmann *et al*., 2013; Zaprasis *et al*., 2015). However, when the cell's demand for proline increases due to osmotic stress, the cell employs the osmo‐adaptive pathway and the ProJ‐ProA‐ProH enzymes (Belitsky *et al*., 2001; Brill *et al*., 2011a). For instance, when *B. subtilis* suffers from severe osmotic upshift, the upregulation of the osmo‐adaptive proline biosynthesis pathway elevates the intracellular proline pools from 20 mM to 500 mM (Whatmore *et al*., 1990; Brill *et al*., 2011a; Hoffmann *et al*., 2013). In *B. subtilis,* the genes encoding for the osmo‐adaptive ProH and ProJ enzymes are genetically organized in the *proHJ* operon. The transcription of the *proHJ* operon is driven by an osmotically controlled promoter, the sequence of which is recognized by the house‐keeping sigma factor of *B. subtilis* SigA (Helmann, 1995; Brill *et al*., 2011b). Notably, the activity of the *proHJ* promoter and the ensuing proline pools have been shown to be linearly dependent on the degree of the osmotic stress imposed onto the cell (Hoffmann *et al*., 2013; Schroeter *et al*., 2013; Godard *et al*., 2020). Hence, monitoring *proHJ* promoter activity and measuring intracellular proline pools can indicate the physiological state of osmotically stressed cells (Hoffmann *et al*., 2017). However, previous studies on the adaptation to osmotic stress and dynamics of the *proHJ* promoter have been based entirely on population‐wide analyses (Brill *et al*., 2011a; Hoffmann *et al*., 2013). Thus, the activation of *proHJ* transcription has always been assessed by averaging the activities of all *B. subtilis* cells in a given culture, thereby missing potential phenotypic variation (heterogeneity) in *proHJ* promoter activity.

Phenotypic heterogeneity is inherent to many regulatory and physiological processes and may arise from a responsive event to specific environmental conditions or from noisy gene expression (Elowitz *et al*., 2002; Kussell, 2005). The ability to switch phenotypes allows microorganisms to survive in challenging surroundings, and most importantly, persist during antibiotic treatments (Balaban *et al*., 2004; Wood *et al*., 2013). Therefore, it is of high relevance to look more in‐depth into *B. subtilis*' response to osmotic stress on a single‐cell level.

In this study, we elucidate the early response of *B. subtilis* to mild osmotic upshift at the single‐cell level by focusing on the transcriptional up‐regulation of *proHJ* proline synthesis genes. Using time‐lapse microscopy, we show heterogeneous *proHJ* transcriptional activation under 0.6 M NaCl treatment and estimate the time needed for cells to adjust to the osmotic upshift and resume cell division. Data presented here show that only cells exceeding a threshold level of *proHJ* expression can efficiently grow and divide under osmotically non‐favourable conditions, whereas those with low expression of *proHJ* do not divide and likely remain dormant. Additionally, we demonstrated that bactericidal antibiotics with different molecular targets as well as direct exposure to hydrogen peroxide significantly upregulate the *proHJ* transcription, in the absence of osmostress, suggesting that the osmotically controlled proline biosynthesis pathway is likely involved in antibiotic ROS‐mediated stress response.

## Results

### Early adaptation to the elevated osmolarity shows increased P*proHJ* activity in *B. subtilis*

During osmotic upshift, *B. subtilis* induces proline biosynthesis to utilize it as a compatible solute in the absence of exogenous osmoprotectants (Brill *et al*., 2011a; Hoffmann *et al*., 2012). To study the adaptation to the hyper‐osmotic environment, we followed the expression of the *proHJ* operon that encodes the osmostress‐adaptive proline biosynthesis enzymes ProH and ProJ. For this purpose, we designed a *B. subtilis* 168 strain with the sfGFP (Sp) gene sequence transcriptionally fused to the *proHJ* promoter with its own RBS and the twenty‐nine codons of *proH*. With the use of this reporter strain, we followed the GFP fluorescent signal, and hence the activity of *proHJ* transcription (Fig. 1A). To ensure that *de novo* synthesized proline was the only available osmoprotectant for *B. subtilis*, all experiments were carried out in SMM minimal media, which possess an osmolarity of 356 mosmol kg^‐1^ (Hoffmann *et al*., 2013), supplemented with 0.5% glucose as a primary carbon source.

**Fig. 2:**
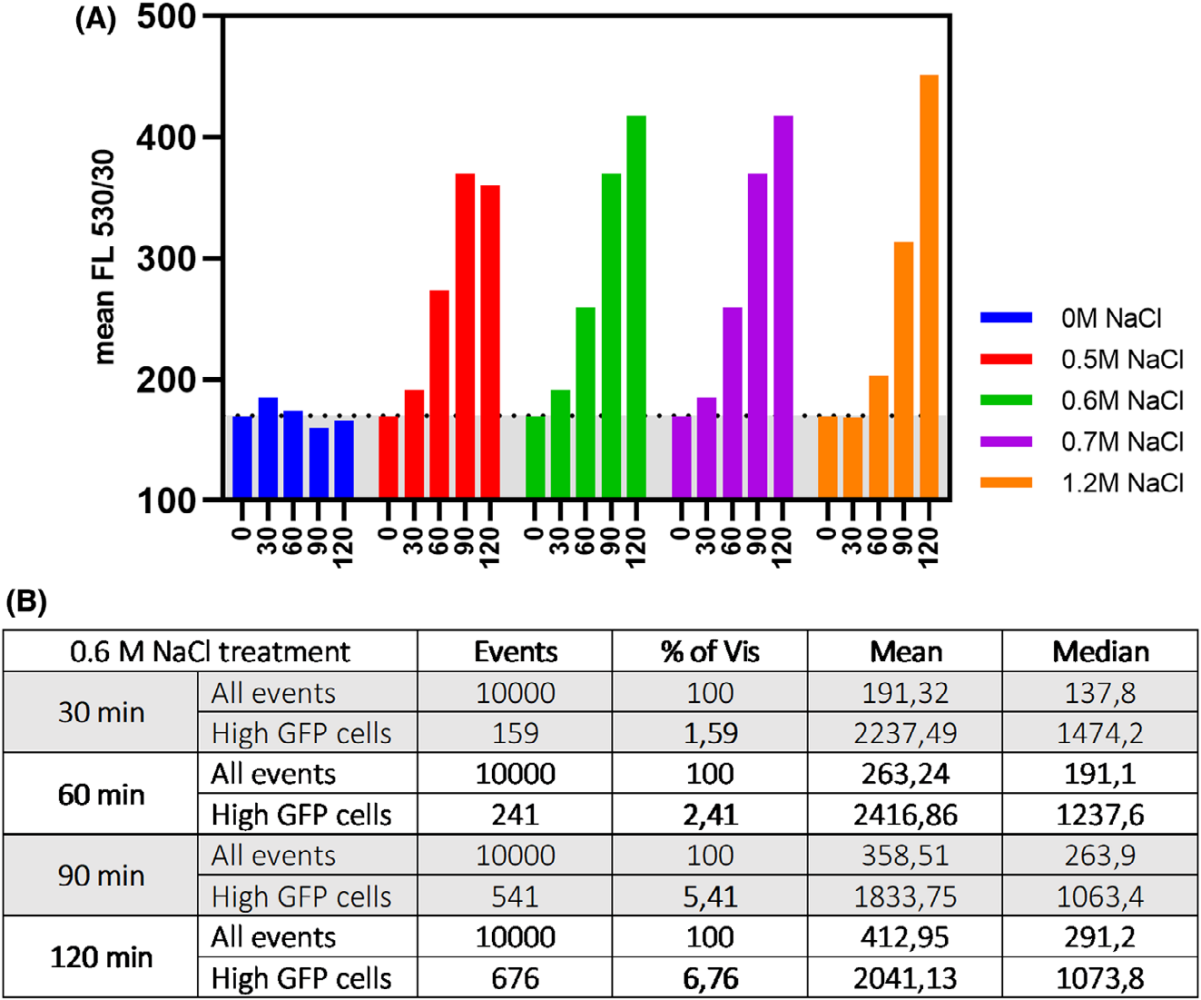
Activation of the *proHJ* promoter upon NaCl exposure. (A) The graph shows the average fluorescence intensity (per cell) of *B. subtilis* 168 P*proHJ*‐*sfGFP (Sp)* during exposure to different NaCl concentrations. The zero‐time point represents the start of NaCl exposure. The dashed line indicates the background GFP signal. The table below (B) shows the population‐wide fluorescence distribution of *B. subtilis* P*proHJ*‐*sfGFP (Sp)* after 30, 60, 90 and 120 min of exposure to 0.6 M NaCl. The total sample size was 10.000 individuals (indicated as events).

First, the early adaptation to the hyperosmotic environment was analysed on a population‐wide scale, and the P*proHJ* activity was measured in cells exposed to different concentrations of sodium chloride (0 M, 0.5 M, 0.6 M, 0.7 M and 1.2 M NaCl) by flow cytometry. The early response to osmotic upshift was analysed for 10,000 cells in each salt concentration at 30 min intervals for 120 min (Fig. 2). Interestingly, we found that the *proHJ* expression was moderately induced within the first 60 min after cells were exposed to hyperosmotic conditions in all samples. After 90 to 120 min of treatment with 0.5 M, 0.6 M, 0.7 M and 1.2 M NaCl, *B. subtilis* cells displayed the highest increase in the average GFP fluorescence in all salt‐treated samples compared to the 0 M NaCl control, indicating the approximate time required for *B. subtilis* to highly induce the *proHJ* expression and thereby attain osmoprotective proline pools (Fig. 2A) (Whatmore *et al*., 1990; Hoffmann *et al*., 2012). These data agree with previous proteomics and transcriptomics studies in *B. subtilis* under high osmotic stress, revealing the highest mRNA levels of *proH* and *proJ* after 60 min of exposure to a high salt concentration (Höper *et al*., 2006; Hahne *et al*., 2010).

Previous studies have shown that osmotically challenged *B. subtilis* increases its intracellular proline concentration to a level that depends on the applied osmotic pressure (Brill *et al*., 2011a; Hoffmann *et al*., 2013; Schroeter *et al*., 2013). Interestingly, the highest variation in the intracellular proline levels was found for cells exposed to mild osmotic stress caused by 0.6 M NaCl (Brill *et al*., 2011a). The flow cytometry data showed that exposure to different salt concentrations resulted in a population‐wide shift in the fluorescence intensity. Although, we did not observe a bimodal distribution of fluorescent signal in any of the cultures pointing on heterogeneous activation of P*proHJ* (Fig. S1), we detected a small fraction of the osmostressed population with a higher fluorescence signal (Fig. 2B). In the case of the 0.6 M NaCl treatment, the fraction of highly fluorescent cells reached 6.8% of the entire population after 120 min of salt exposure, which likely resulted from the stochastic activation of the promoter (Fig. 2B). These results show that under mild osmostress conditions, a part of the population highly induces *proHJ* expression in order to synthesize large amounts of the compatible solute proline, whereas the majority of the population maintains moderate levels of *proHJ* expression (Whatmore *et al*., 1990; Brill *et al*., 2011a). The variation in the intracellular proline concentration in the 0.6 M NaCl‐treated cells (Brill *et al*., 2011a; Hoffmann *et al*., 2017) and the presence of a fraction of highly fluorescent cell variants shown by the flow cytometry data suggests that under these conditions, the population of genetically identical *B. subtilis* cells diversifies in terms of the activation of the proline biosynthesis pathway.

### Upregulation of *proHJ* transcription on a single‐cell level during osmotic upshift – heterogeneous activation of the proline biosynthesis pathway

**Fig. 3:**
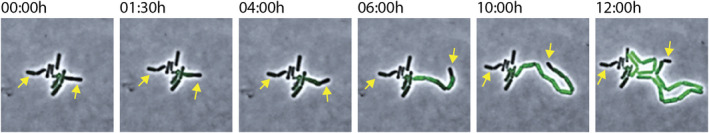
Time‐lapse images of *B. subtilis* P*proHJ*‐*sfGFP (Sp)* exposed to 0.6 M NaCl showing heterogeneity in *proHJ* transcription. Micrographs of time‐lapse microscopy showing adaptation of *B. subtilis* to 0.6 M NaCl exposure at indicated time points. Only one cell becomes fluorescent after an initial lag time and resumes growth and division. The non‐fluorescent cells, marked with yellow arrows, could not divide and remain dormant throughout the 12 h of exposure to sustained osmostress.

To further investigate *B. subtilis'* adaptation to 0.6 M NaCl stress, we performed a time‐lapse microscopy experiment on SMM agarose containing 0.6 M NaCl and monitored P*proHJ* activation at a single‐cell level. Interestingly, time‐lapse microscopy revealed that under sustained osmotic upshift, *B. subtilis* responds heterogeneously and only part of the isogenic population highly activates *proHJ* transcription (Fig. 3). After the first 90 min of exposure to the osmotic upshift, cells did not grow nor divide, and initially, only a fraction of the population displayed a low GFP signal, indicating moderate P*proHJ* activity and upregulation of the proline biosynthesis pathway (Fig. 3).

Notably, after an initial lag phase of 6 h, GFP‐positive cells resumed growth and division and gradually increased GFP signal over time, indicating increased P*proHJ* activity and high upregulation of the proline biosynthesis pathway (Fig. S2). Conversely, GFP‐negative cells did not show the P*proHJ* activation and could not divide. This data suggests that GFP‐positive cells were more adapted to elevated osmotic pressure by effectively switching on the proline biosynthesis pathway and reaching for the accumulated proline pools to balance out the adverse effects of the osmotic upshift. Based on these results, we propose that the cell needs to exceed a particular intracellular proline concentration, which allows it to overcome the detrimental effects of water efflux and thereby permits the resumption of growth and division.

### Changes in regulation of the osmostress‐adaptive proline biosynthesis pathway upon antibiotic stress

We reasoned that a short pretreatment with an osmotic upshift (0.6 M NaCl) can benefit the *B. subtilis* population and helps to increase its survival upon aminoglycoside exposure. Thus, we wondered whether *B. subtilis* activates the osmostress‐adaptive proline biosynthesis pathway in response to immediate antibiotic treatment on a population‐wide level. To address this question, we followed the activation of the *proHJ* promoter under antibiotic exposure in cultures with and without the 90 min of pretreatment with an osmotic upshift. Accordingly, to determine the kinetics of P*proHJ*, we distributed differently adapted precultures of *B. subtilis* to 96‐well microtitre plates, and we followed the optical density and the GFP fluorescent signal across a broad range of kanamycin concentrations for 16 h.

**Fig. 4:**
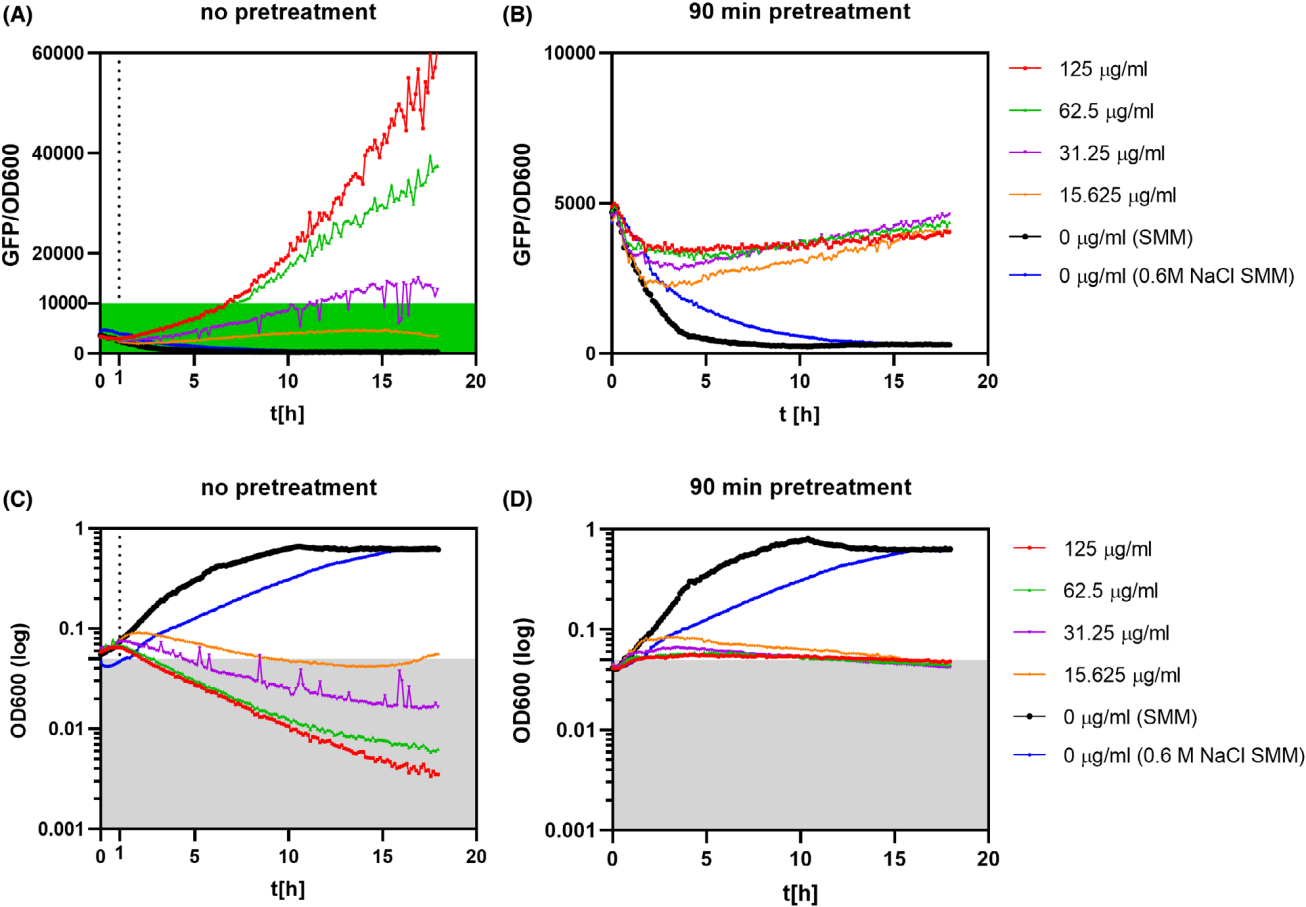
Activation of P*proHJ‐sfGFP (Sp)* under exposure to kanamycin with or without osmostress pre‐treatment. After reaching OD600 0.35, the culture was split into two equal volumes where one part was subjected to 0.6 M NaCl for 90 min, and the second one was kept in the neutral osmolarity SMM. After the preadaptation step, cultures were washed in a fresh SMM medium without NaCl and distributed to the 96‐well plate containing increasing concentrations of kanamycin. (A) and (B) represent changes of the relative fluorescent units in time of differently preadapted *B. subtilis* cultures in the presence of increasing concentrations of kanamycin (0–125 µg ml^−1^). (C) and (D) show corresponding growth curves of cultures without and with 90 min 0.6 M NaCl pretreatment. The black curve indicates the P*proHJ* activity of cultures without kanamycin for each experiment. The blue line indicates cultures that were continuously exposed to 0.6 M NaCl. The dotted line indicates the increase in cell lysis. All measurements were performed in biological triplicates, and the mean values were plotted on the graphs. The relative fluorescent signal was estimated by dividing the GFP signal by the cell density. The value is represented in arbitrary units.

Interestingly, the GFP expression data revealed significant upregulation of the *proHJ* transcription for cultures exposed solely to kanamycin treatment (Fig. 4A). Notably, we found that the level of P*proHJ* activation in cells directly exposed to kanamycin was mostly antibiotic dose‐dependent, and the relative GFP fluorescence significantly increased in time when the antibiotic concentrations exceeded the MIC (15.625 µg ml^−1^). The high fluorescence values of kanamycin‐treated cells correlated with the substantial loss of viability, presumably due to the antibiotic‐mediated cell damage. (Fig. 4C). This suggests that either the remaining persisting cells highly activated the *proHJ* transcription or P*proHJ* is highly activated upon induced cell lysis. Moreover, in agreement with our previous study, cultures pre‐treated with salt were more resistant towards kanamycin and the growth inhibition profiles indicated growth stasis, typically observed for cells exposed to bacteriostatic antibiotics (Fig. 4D). The non‐growing but metabolically active cells displayed significantly reduced P*proHJ* activity when exposed to the same concentrations of kanamycin as actively growing cells (Fig. 4A, B). However, despite the metabolic changes and growth inhibition, antibiotic‐treated cells exhibited continuous and moderate expression of *proHJ* (Fig. 4B).

Taken together, these data suggest that the osmotically controlled proline biosynthetic pathway is activated not only in response to osmotic or cold stress (Brill *et al*., 2011a; Hoffmann and Bremer, 2011; Hoffmann *et al*., 2017) but also in response to antibiotic stress. Moreover, we show that cells pre‐treated with 0.6 M NaCl for 90 min prior to the antibiotic treatment likely reach for proline resources to counteract the antibiotic stress directly.

### Activation of P*proHJ* by bactericidal antibiotics reveals different levels of *proHJ* transcription

**Fig. 5:**
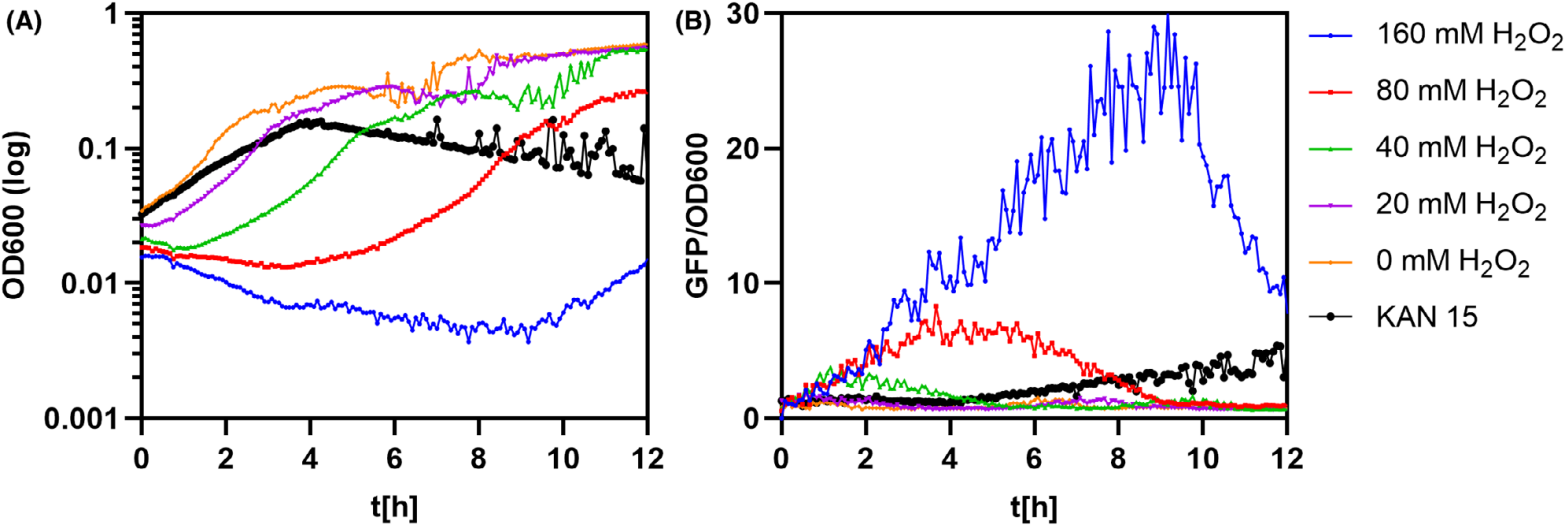
Oxidative stress activates the *proHJ* transcription in *B. subtilis* 168. Exponentially growing cultures in SMM were exposed to sub‐lethal concentrations of hydrogen peroxide and incubated for 12 h at 37°C. (A) shows the differences in optical densities, whereas (B) represents the relative fluorescent GFP signal measured from the corresponding cultures. The orange curve represents the outgrowth and relative fluorescent signal of control samples without a stressor. The black curve represents the outgrowth and relative fluorescent signal of control samples with MIC of kanamycin. All measurements were performed in biological duplicates, and the mean values were plotted on the graphs. The relative fluorescent signal was estimated by dividing the GFP signal by the cell density. The value is represented in arbitrary units.

Bactericidal antibiotics display various modes of action by interfering with diverse molecular targets. For instance, aminoglycosides target 30S ribosomal subunit and consequently impair mRNA translation (Magnet and Blanchard, 2005; Borovinskaya *et al*., 2007), whereas fluoroquinolones act on the DNA level and perturb DNA replication (LeBel, 1988). However, recent studies have pointed to a common mechanism of antibiotic‐mediated cell death, which involves increased production of hydroxyl radicals via the TCA cycle and iron metabolism (Kohanski *et al*., 2007; Dwyer *et al*., 2009). Consequently, the increased level of reactive species causes oxidative stress damage of the cellular components, and eventually, cell lysis. Moreover, the differences in bactericide's lethality have been correlated with the level of ROS‐mediated oxidative damage (Dwyer *et al*., 2009, 2014). Since proline is known to act as a molecular chaperon and ROS scavenger (Trelstad *et al*., 1981; Kaul *et al*., 2008), we hypothesize that *B. subtilis* activates the *de novo* proline biosynthesis pathway as a stress response towards antibiotic‐induced oxidative stress. Firstly, to test our hypothesis, we examined the role of oxidative stress on P*proHJ* kinetics and followed the *proHJ* activation on a population‐wide level in cells challenged with sub‐lethal concentrations of hydrogen peroxide. Secondly, prompted by the kanamycin dose‐dependent *proHJ* activation data (Fig. 5), we asked if exposure to other bactericidal antibiotics, which induce ROS formation, activate the *de novo* proline biosynthesis pathway.

Interestingly, the GFP expression data showed that direct exposure to hydrogen peroxide also activates the *proHJ* transcription in *B. subtilis*. The fluorescent signal from the reporter strain showed an increased GFP signal when cells were exposed to increasing concentrations of hydrogen peroxide (Fig. 5B). Moreover, we observed that the activity of the *proHJ* promoter decreased when cells regained their growth rate, suggesting that the activation of *proHJ* transcription and proline synthesis contributes to adaptation to oxidative stress.

**Fig. 6:**
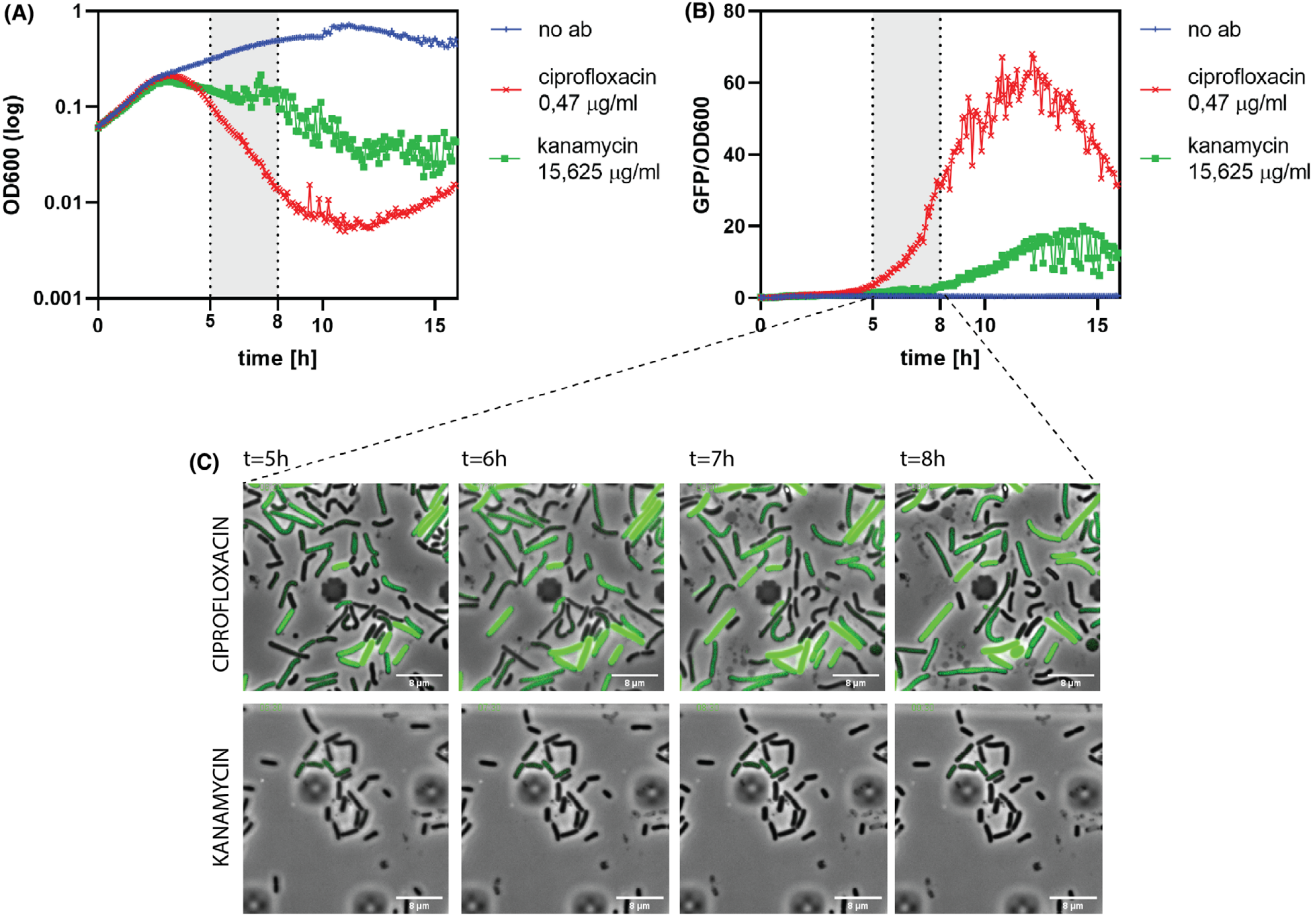
Exposure to bactericides highly activates the *proHJ* transcription in *B. subtilis* 168. Exponentially growing cultures in SMM were treated with the MIC of kanamycin (green curve) and ciprofloxacin (red curve) and incubated for 16 h at 37°C. (A) shows the differences in optical densities of tested cultures, whereas (B) represents the relative fluorescent GFP signal measured from the corresponding cultures. The grey‐shaded area indicates the time frame when micrographs were taken (C). The blue curve represents the outgrowth of control samples without antibiotic treatment. All measurements were performed in biological duplicates, and the mean values were plotted on the graphs. The relative fluorescent signal was estimated by dividing the GFP signal by the cell density. The value is represented in arbitrary units.

Next, to compare the levels of P*proHJ* activation in cells exposed to different bactericidal antibiotics, we decided to use MIC of kanamycin (15,625 µg ml^−1^) and ciprofloxacin (0,47 µg ml^−1^) as study conditions. The analysis of fluorescent data revealed that fluoroquinolones activate the *PproHJ* significantly higher than aminoglycosides (Fig. 6B). Based on the significant differences in the relative fluorescence between kanamycin and ciprofloxacin‐treated cultures, we propose that cells exposed to antibiotics of different classes, which interfere with different primary molecular targets, induce the *proHJ* transcription to different extents.

Furthermore, to confirm the population‐wide fluorescence data and characterize antibiotic‐treated cells on a single‐cell level, we designed a time‐lapse microscopy experiment and used a microfluidics device, which allowed us to apply different growth conditions to follow the cell growth and the fluorescent signal of individual cells in real‐time. In agreement with the previous fluorescence measurements (Fig. 6B), the analysis of microscopy data confirmed that *B. subtilis*' cells exposed to different bactericidal antibiotics activate the *proHJ* promoter on different levels (Fig. 6C). Moreover, we observed differences in phenotypes of cells exposed to kanamycin compared to those exposed to ciprofloxacin. In the case of kanamycin treatment, we identified a non‐growing population in which only part of the cells exhibited a moderate GFP fluorescence within 8 h of antibiotic exposure. In contrast, the ciprofloxacin‐treated population exhibited a high cell‐to‐cell variation in the GFP expression and severe cell elongation followed by rapid lysis. Because the exposure to ciprofloxacin resulted in elongated cells, we suggest that the observed differences in the *proHJ* activity between kanamycin and ciprofloxacin treatments might not only result from ROS formation but also from changes in the cell's turgor.

### The antibiotic‐mediated *proHJ* induction is independent of surfactant‐induced membrane perturbations

Kanamycin and ciprofloxacin belong to the bactericidal group of antibiotics known to indirectly induce cell death by ROS‐mediated lipid peroxidation and, in consequence, membrane damage (Dwyer *et al*., 2009, 2014). It could be argued that the induction of *proHJ* transcription with antibiotics could be a consequence of antibiotic‐induced leakage of the cell membrane and loss of turgor. Hence, we tested if direct perturbation of the cell's envelope by membrane‐acting agents can activate the *proHJ* transcription. Rhamnolipids are commonly used glycolipid biosurfactants that sensitize Gram‐positive and Gram‐negative bacteria to aminoglycosides by forming membrane pores and facilitating antibiotic's uptake (Radlinski *et al*., 2019). Therefore, we used a sub‐lethal concentration of rhamnolipids (15 µg ml^−1^) to induce ROS‐independent membrane damage and follow the P*proHJ* activation.

**Fig. 7:**
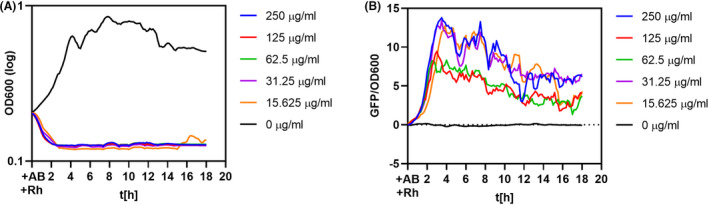
Perturbations of the cell membrane do not induce the *proHJ* transcription in *B. subtilis* 168. Exponentially growing cultures in SMM were exposed to the sub‐MIC (15 µg ml^−1^) of mono‐ and di‐rhamnolipid congeners. The cells were treated with or without kanamycin and incubated for 18 h at 37°C. (A) shows the differences in optical densities of tested cultures, whereas (B) represents the corresponding relative fluorescent GFP signal. The black curve shows the OD600 and RFU for cells treated only with rhamnolipids. All measurements were performed in biological duplicates, and the mean values were plotted on the graphs. The relative fluorescent signal was estimated by dividing the GFP signal by the cell density. The value is represented in arbitrary units.

Accordingly, we examined the level of the *proHJ* upregulation in cells with and without kanamycin treatment in the presence of a sub‐lethal concentration of rhamnolipids (Fig. 7). The fluorescent data revealed that cells exposed solely to rhamnolipids did not activate the *proHJ* transcription. Conversely, cells treated with a mix of rhamnolipids and kanamycin displayed strong upregulation of the *proHJ* promoter (Fig. 7B). This evidence demonstrates that the induction of the proline biosynthetic pathway in antibiotic‐stressed *B. subtilis* is independent of directly induced membrane damage.

### Exogenous proline decreases the intracellular ROS levels and benefits the survival under antibiotic stress

*B. subtilis* imports proline directly from the media via two transporter systems, OpuE and PutP (von Blohn *et al*., 1997; Moses *et al*., 2012). While PutP is primarily responsible for the uptake of proline when it is used as a nutrient (Moses *et al*., 2012), OpuE primarily serves for proline import when it is used as an osmostress protectant (von Blohn *et al*., 1997). Here, we tested if the addition of proline to the media would increase the survival of *B. subtilis* when exposed to lethal doses of kanamycin. Moreover, to examine the role of proline in oxidative stress response and ROS scavenging, we estimated intracellular ROS levels in antibiotic‐stressed *B. subtilis* with and without proline addition. To follow the changes in the ROS pools, we used 2',7'‐dichlorodihydrofluorescein diacetate (H_2_DCFDA), a chemically reduced form of fluorescein. Inside the cell, H_2_DCFDA is cleaved by intracellular esterases and oxidized in the presence of hydroxyl radicals to the highly fluorescent 2',7’‐ dichlorofluorescein (DCF). Thus, the conversion of H_2_DCFDA to DCF directly corresponds to the ROS levels.

**Fig. 8:**
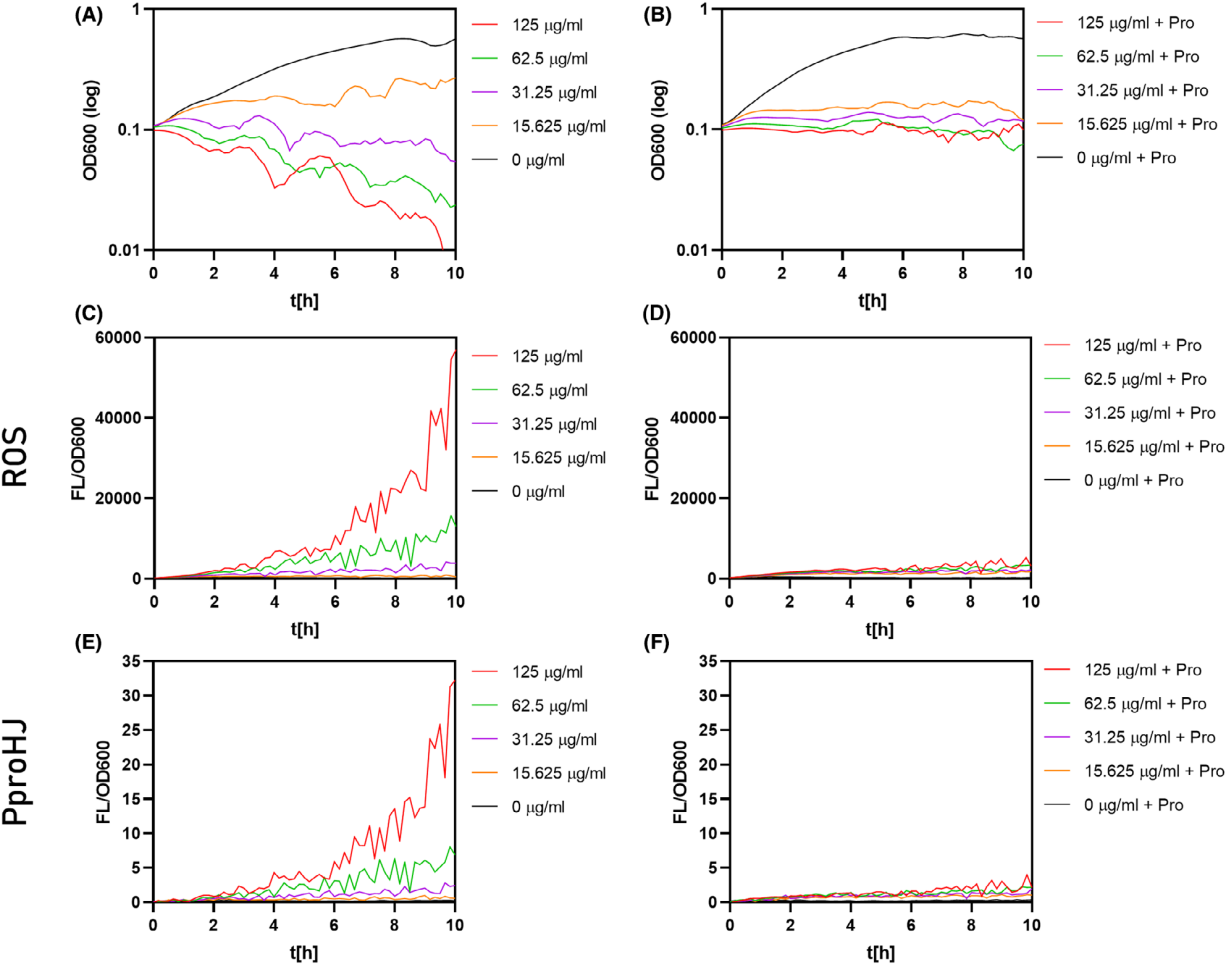
Protective effect of exogenous proline during kanamycin stress and intracellular ROS levels. Exponentially growing cultures in SMM were treated with increasing kanamycin concentrations together with (B, D, F) or without (A, C, E) 40 mM of proline in SMM medium and incubated for 10 h at 37°C. (A) and (B) show the growth inhibition curves for cells without and with proline supplementation. (C) and (D). represent the relative fluorescent signal from dichlorofluorescein measured from the corresponding cultures. (E) and (F) show the activity of the P*proHJ* promoter, in this case, we used P*proHJ*‐*tagRFP* reporter strain to follow simultaneously the ROS formation and P*proHJ* activity. The black curve represents the outgrowth of control samples without antibiotic treatment. All measurements were performed in biological triplicates, and the mean values were plotted on the graphs. The relative fluorescent signal was estimated by dividing the fluorescent signal by the cell density. The value is represented in arbitrary units.

Interestingly, the presence of exogenous 40 mM proline positively affected the growth of kanamycin‐stressed cells above the MIC values (Fig. 8A and C). Compared to the control, the cells supplemented with proline displayed significantly lower activation ROS levels when exposed to kanamycin (Fig. 8C and D). Moreover, we were able to simultaneously follow the P*proHJ* activation under tested conditions, and the P*proHJ* activity elegantly correlates with the ROS levels (Fig. 8E and F). These evidences show that proline has antioxidative potential and the *proHJ* promoter is activated by ROS. The decrease in the P*proHJ* in cells with additional proline suggests that the demand for the ProH‐ and ProJ‐dependent proline synthesis decreased when the proline was repurposed from the media.

## Discussion

In the absence of external sources of compatible solutes, *de novo* synthesis and accumulation of proline play a pivotal role in *B. subtilis*' adaptation to an adverse hyperosmotic pressure (Whatmore *et al*., 1990; Brill *et al*., 2011a). In this study, we followed for the first time the response of *B. subtilis* to the osmotic upshift and activation of the *proHJ* transcription on a single‐cell level, using P*proHJ*‐*sfGFP* (Sp) as reporter strain.

Examining the early response to osmotic upshift with flow cytometry revealed that 60–90 min of NaCl exposure is sufficient to induce *proHJ* transcription and activate the osmotically induced proline biosynthesis in *B. subtilis*, which is in agreement with the results of related studies (Höper *et al*., 2006; Hahne *et al*., 2010; Brill *et al*., 2011a) (Fig. 2). Additionally, we have shown that the *proHJ* promoter is upregulated in the majority of the population and confirmed that the level of its activation depends on the severity of the osmotic upshift (Brill *et al*., 2011a; Schroeter *et al*., 2013; Hoffmann *et al*., 2017). In the heterogeneous model system, where *proHJ* transcription can be either on or off, a bimodal fluorescence distribution amongst the cell population may be expected. However, the flow cytometry data showed no clear bimodal distribution, but only the presence of a small fraction of highly fluorescent cells (Fig. S1). To complement the flow cytometry data, we were able to show substantial heterogeneity in the activation of the *proHJ* operon at 0.6 M NaCl using time‐lapse microscopy. We demonstrated that under mild osmotic upshift, the clonal population of *B. subtilis* diversifies into two distinct subpopulations: the *proHJ* inducers and non‐inducers. We showed that cells that activated the *proHJ* transcription and consequently increased intracellular proline pools were able to resume their pre‐shock growth rates, whereas those that did not show the significant upregulation of the *proHJ* promoter remained dormant (Fig. 3).

It should be noted that the growth conditions within the microcolonies on the agarose pad are different compared to the conditions in liquid media. When growing on solid agarose‐based media, the availability of nutrients in the nearest surroundings decreases over time but also, the concentration of secreted compounds increases. Therefore, we hypothesize that cells that highly induce the *proHJ* expression and boost proline biosynthesis might aid the neighbouring *proHJ* non‐inducers to withstand the osmotic upshift. Since proline can be imported via OpuE (von Blohn *et al*., 1997) and PutP (Moses *et al*., 2012) transporter systems, it would be interesting to examine if the *proHJ* non‐inducers recapture proline excreted to the media (Hoffmann *et al*., 2012). Release of proline by cells that strongly upregulate the *proHJ* promoter and its recapture by the *proHJ* non‐inducers might reflect the cooperative behaviours, similar to those reported for the glycine betaine transport in *Vibrio cholerae* (Kapfhammer *et al*., 2005). The preliminary experiments with *opuE* and *putBCP* mutants showed that cells strongly induce the *proHJ* operon compared to the wild‐type strain after 90 min of the osmotic upshift, and the fraction of cells with high GFP signal substantially increased compared to the wild‐type strain (82% for ∆*opuE* and 63% for ∆*putBCP*) (Fig. S3). This suggests that limited proline recapture (mainly due to the *opuE* deletion) forces cells to increase the *proHJ* transcription in most of the cells in the population (Fig. S3).

So far, it has been reported that in *B. subtilis,* the *proHJ* promoter is induced in response to ionic and non‐ionic solutes (Brill *et al*., 2011b) and cold stress (Hoffmann and Bremer, 2011). However, the transcriptomic study of Nicolas *et al*. (2012) revealed increased levels of the *proHJ* operon transcript under several other conditions, including sporulation, heat stress, glucose starvation and hydrogen peroxide stress (Nicolas *et al*., 2012). Here, for the first time, our population‐wide study showed that the *proHJ* promoter is activated in response to bactericidal antibiotics like kanamycin and ciprofloxacin, (Figs 4 and 6) and confirmed the *proHJ* upregulation under hydrogen peroxide stress (Fig. 5). Interestingly, studies of bulk cultures combined with microscopy analysis revealed that fluoroquinolones have a more substantial effect on the P*proHJ* activation compared to aminoglycosides (Fig. 6). Aminoglycosides and fluoroquinolones share a common killing mechanism that involves ROS‐mediated damage of proteins, lipids and DNA (Dwyer *et al*., 2009, 2014). Since we have shown the increased P*proHJ* activity in cells treated with a non‐radical oxidant (Fig. 5) and direct correlation of the promoter’s activity with ROS levels (Fig. 8), we propose that *B. subtilis* activates *de novo* proline biosynthesis in response to antibiotic‐induced ROS formation and oxidative stress. The differences in the level of *proHJ* activation in cells treated with kanamycin and ciprofloxacin could be explained by differences in primary molecular targets of tested antibiotics and the level of induced ROS (Dwyer *et al*., 2014). In this context, it is worth to note that ciprofloxacin is an inhibitor of gyrase, an enzyme that relaxes the degree of DNA supercoiling. High osmolarity triggers an increase in DNA supercoiling and thereby induces transcription of osmostress‐ responsive genes (Higgins *et al*., 1988; Cheung *et al*., 2003).

**Fig. 9:**
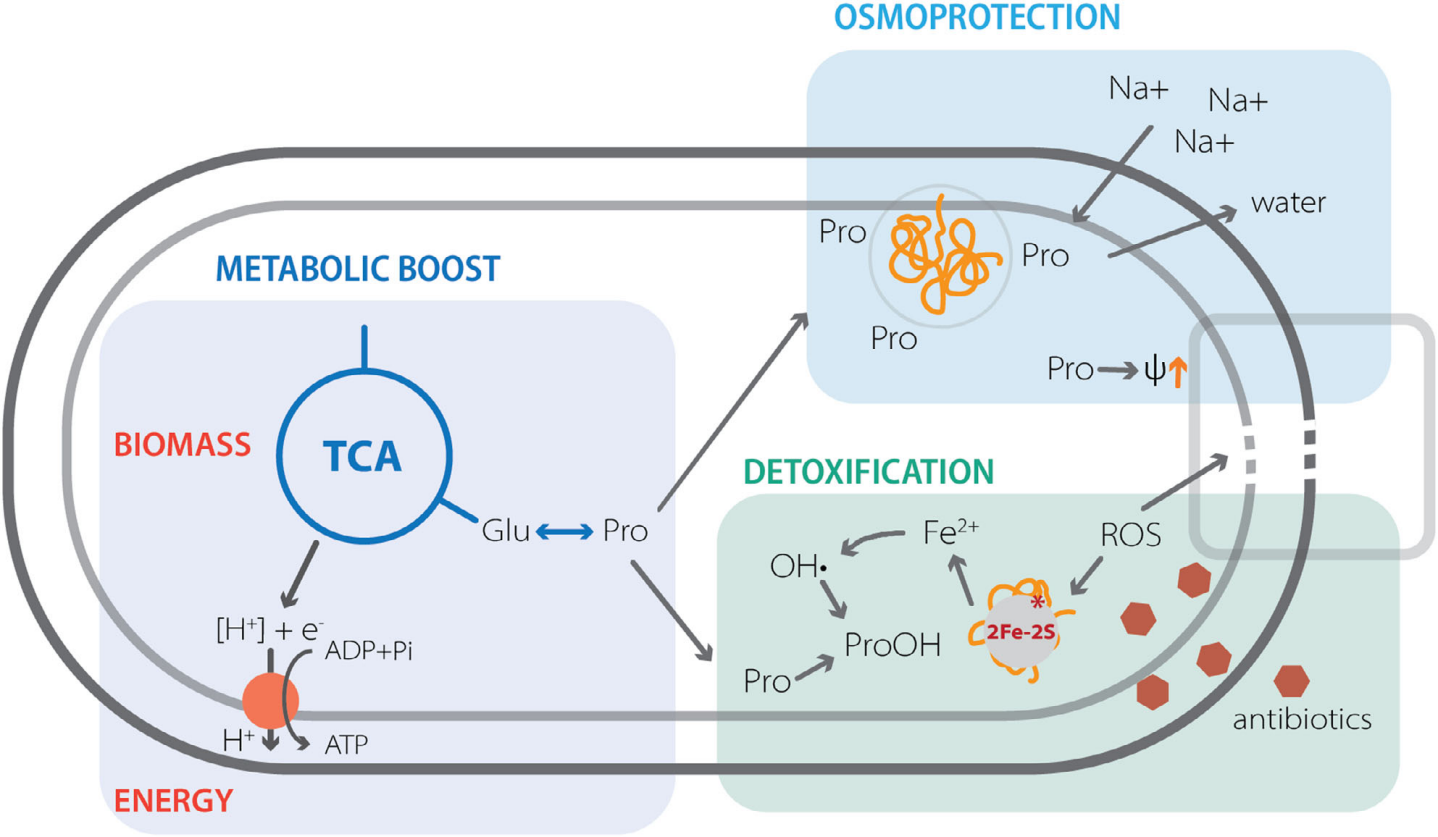
Schematic representation of proline roles in *B. subtilis*. Overview of the proline contributions in osmoprotection, ROS detoxification and energy production.

But why *B. subtilis* induces the genes for the osmostress‐responsive *de novo* proline biosynthesis pathway when suffering from the antibiotic and oxidative stress? Except for its osmoprotective properties, proline has a vital role in overcoming oxidative stress by directly reacting with hydroxy radicals and by forming for the cell harmless proline derivatives (Floyd and Nagy, 1984; Natarajan *et al*., 2012; Liang *et al*., 2013). Since the bactericidal mode of action includes ROS formation and oxidative stress damage of the membranes, we believe that in *B. subtilis,* the upregulation of the *proHJ* promoter and increased proline biosynthesis not only offsets turgor imbalances but also aids with adverse effects of ROS (Fig. 9). To support our hypothesis, we have shown increased survival and substantially decreased ROS levels of *B. subtilis* cells upon kanamycin stress with 40 mM proline in the media (Fig. 8), suggesting a supporting role of proline in antibiotic‐mediated oxidative stress response.

Interestingly, Wong *et al*. (2021) recently proposed that aminoglycosides and fluoroquinolones mediate cell death and lysis by inducing cytoplasmic condensation through ROS‐induced membrane damage (Wong *et al*., 2021). Because cytoplasmic condensation and lysis are associated with loss of cellular turgor (Wong and Amir, 2019), the cell needs to synthesize sufficient amounts of osmoprotectants to counteract the high ionic strength of cytoplasm. Notably, we show that *B. subtilis* cells activate the *proHJ* promoter in response to the antibiotic‐induced cell damage but not to the surfactant‐induced membrane damage (Fig. 7), indicating that artificially‐induced membrane damage and relative loss of turgor are not sufficient to induce *de novo* proline biosynthesis. We propose that *B. subtilis* activates *de novo* proline biosynthesis primarily in direct response to increased ROS levels and secondarily due to the ROS‐mediated membrane damage and increased ionic strength of the cytoplasm.

To conclude, our study sheds new light on the activation of *de novo* proline biosynthesis pathway in osmotically stressed *B. subtilis* on a single‐cell level and its possible role in antibiotic‐mediated cell damage. Based on our findings, we propose that proline has an essential role, not only in maintaining the cell turgor, but also in ROS‐detoxification; however, the exact mechanism has yet to be revealed in future studies.

## Experimental procedures

### Bacterial strains, culture conditions and media

All tested strains used in this study are derivatives of the *B. subtilis* 168 *trpC2* laboratory strain (Table S1). First transformation steps were performed in the initial hosts, *E. coli* MC1061. Molecular cloning was performed as described by Sambrook *et al*. (1989). Used strains were cultured at 37°C with aeration, initially in liquid Luria Bertani media (LB) or agar LB, with 5 μg ml^−1^ chloramphenicol for engineered *B. subtilis* strains or 100 μg ml^−1^ ampicillin for *E. coli* strains. Further on, *B. subtilis* strains were grown in Spizizen's Minimal Medium (Harwood and Cutting, 1990) (SMM) supplemented with 0.5% (w/v) glucose, 1% (v/v) trace elements and, for tryptophan‐autotroph strains, 0.5% (w/v) tryptophan. To maintain the same culture conditions, 3‐day cultivation was performed as follows: *B. subtilis* was streaked on selective LB agar plates and incubated overnight (O/N) at 37°C. The next morning, 3 ml of liquid LB was inoculated with a single colony and incubated for 8 h at 37°C and 220 rpm in a shaking incubator. After the incubation period, the culture was diluted 1:1000 in supplemented SMM medium and grown O/N at 37°C and 220 rpm. The overnight culture was diluted to OD600 of 0.08 in fresh SMM medium and grown till the OD600 reached 0.3. The exponentially growing culture was immediately subjected to osmotic stress, and the following measurements were taken.

### Construction of a *B. subtilis* 168 P*proHJ*‐*sfGFP* (Sp) reporter fusion

A promoter P*proHJ*‐sfGFP fusion was made to follow the dynamics of *proHJ* genes expression at the single‐cell level during osmotic stress. A 934 bp region with the *proHJ* promoter and first 29 codons of *proHJ* was transcriptionally fused with super folder GFP (Sp) and the plasmid was integrated into *B*. *subtilis* 168 strain via Campbell‐type single crossover integration.

### Flow cytometry

FACS measurements were performed using a BD Biosciences FACS Canto Flow Cytometer. FACS was used to determine the fluorescence of exponentially growing *B. subtilis* P*proH‐sfGFP* (Sp) after 30, 60, 90 or 120 min of exposure to 0.5 M, 0.6 M, 0.7 M or 1.2 M NaCl. FACS samples were diluted 1000‐fold in phosphate‐buffered saline (PBS, Boom (Oxoid, Meppel, The Netherlands)) before measurement.

### Microtitre‐plate experiments

Bacterial antibiotic resistance and fluorescent measurements were carried in either Varioskan™ LUX (Thermofisher, Waltham, MA, USA) or Tecan T200 (Tecan, Männedorf, Switzerland) multimode microplate reader. (An exponentially growing culture was exposed to 0.6 M NaCl for 90 min, diluted to an OD600 of 0.1 in SMM, and diluted 2‐fold in antibiotic‐containing SMM. Final microtitre plate volume was 135 μl per well. Cells were grown for a total of 16 h, unless noted otherwise.

### Reactive oxygen species measurements

ROS levels were measured using 10 µM of 2’,7’‐dichlorofluorescein diacetate (H_2_DCFDA) fluorescent dye. All measurements were performed in 96‐well black microtitre plates using Varioskan™ LUX multimode microplate reader (Thermofisher). An exponentially growing culture was diluted to an OD600 of 0.1 in fresh SMM and exposed to antibiotics. Final microtitre plate volume was 135 μl per well. OD600 was measured every 10 min over 10 h. Three independent experiments were performed for each condition. Fluorescence was measured with excitation at 488 nm and emission at 510 nm. The control without H_2_CDFDA was used to correct for autofluorescence. As additional control, we used 0.05% N‐Acetylcysteine (NAC) antioxidant.

### Fluorescence time‐lapse microscopy

Fluorescence time‐lapse microscopy was performed using an Olympus IX71 DeltaVision microscope. SMM‐agarose microscope slides were prepared with 1.5% (w/v) Low Melting Point Agarose (Sigma‐Aldrich, St. Louis, MO, USA) and optionally kanamycin or NaCl. Exponentially growing cells were either exposed to 0.6 M NaCl for 90 min and applied to an agarose slide or directly applied to an agarose slide containing 0.6 M NaCl. Cells were tracked by imaging phase‐contrast and fluorescence every 12 min over 10 h. Image processing was done with ImageJ version 1.50 b software.

### Microfluidics experiments

The experiments were performed with the CellASIC ONIX microfluidic device (Merck Millipore, Darmstadt, Germany) in a B04A plate. The plate was primed with the medium before the experiment, using the manufacturer's specifications. A pump pressure of 0.25 psi drove the flow rate of the supplied medium. Exponentially growing cells were diluted in the complete SMM to OD600 0.05 and subsequently loaded on the microfluidics plate. The microfluidic plate was mounted on an Olympus IX71 DeltaVision microscope, and fluorescence time‐lapse microscopy was performed. The changes of growing conditions in each chamber were performed accordingly: 90 min regrowth in SMM and 8 h of antibiotic treatment. Cells were tracked by imaging phase‐contrast and fluorescence every 10 min over 12 h. All experiments were performed at 37°C.

## Conflict of interest

None declared.

## Supporting information


**Fig. S1**. Activation of the *proHJ* promoter in response to the osmotic upshift. Fluorescence distribution of *B. subtilis* 168 P*proHJ*‐*sfGFP (Sp)* strain in response to the different osmolarity of the environment after 90 min. The total sample size was 10.000 individual cells.
**Fig. S2**. Differences in the *proHJ* expression from dividing and non‐dividing cells. The mean GFP fluorescent signal of dividing and non‐dividing *B. subtilis* cells during 12 h of exposure to the sustained osmotic upshift. 1.5 h' time‐point, indicated with a dotted line, shows the mean FL value for the early response to the osmotic upshift. The 6 h' time‐point marks the beginning of the GFP‐positive cells division. The mean FL was measured with the MicrobeJ plugin for ImageJ, and the error bars show the SD of the measured signal dividing and non‐dividing cells from a single frame.
**Fig. S3**. Activation of the *proHJ* promoter in osmotically stressed *B. subtilis* mutants. (A) Estimation of the average intensity of GFP fluorescent signal and (B) the distribution of the GFP fluorescent signal in studied populations. The percentages in the brackets indicate the exact percentages of cells exhibiting high fluorescent signal. (C) Micrographs of time‐lapse microscopy showing adaptation of different mutants of *B. subtilis* to 0.6 M NaCl exposure at 90 min. The GFP fluorescent signal represents the activity of the *proHJ* promoter. The bar scale indicates 10 µm.
**Table S1**. Strains used in this study.
**Table S2**. Plasmids used in this study.
**Table S3**. Oligonucleotides used in this study.
**Table S4**. Media and buffers.
