# Supplementary material for: Stress‐induced activation of the proline biosynthetic pathway in Bacillus subtilis: a population‐wide and single‐cell study of the osmotically controlled proHJ promoter
Source: Microb Biotechnol. 2022 May 20;15(9):2411–25. doi: 10.1111/1751-7915.14073 (PMC9437891; doi:10.1111/1751-7915.14073)
Supplement: Supplementary file 1 — Fig. S1. Activation of the proHJ promoter in response to the osmotic upshift. Fluorescence distribution of B. subtilis 168 PproHJ‐sfGFP (Sp) strain in response to the different osmolarity of the environment after 90 min. The total sample size was 10.000 individual cells. Fig. S2. Differences in the proHJ expression from dividing and non‐dividing cells. The mean GFP fluorescent signal of dividing and non‐dividing B. subtilis cells during 12 h of exposure to the sustained osmotic upshift. 1.5 h' time‐point, indicated with a dotted line, shows the mean FL value for the early response to the osmotic upshift. The 6 h' time‐point marks the beginning of the GFP‐positive cells division. The mean FL was measured with the MicrobeJ plugin for ImageJ, and the error bars show the SD of the measured signal dividing and non‐dividing cells from a single frame. Fig. S3. Activation of the proHJ promoter in osmotically stressed B. subtilis mutants. (A) Estimation of the average intensity of GFP fluorescent signal and (B) the distribution of the GFP fluorescent signal in studied populations. The percentages in the brackets indicate the exact percentages of cells exhibiting high fluorescent signal. (C) Micrographs of time‐lapse microscopy showing adaptation of different mutants of B. subtilis to 0.6 M NaCl exposure at 90 min. The GFP fluorescent signal represents the activity of the proHJ promoter. The bar scale indicates 10 µm. Table S1. Strains used in this study. Table S2. Plasmids used in this study. Table S3. Oligonucleotides used in this study. Table S4. Media and buffers. [file MBT2-15-2411-s001.docx]

# SUPPLEMENTARY MATERIALS

| 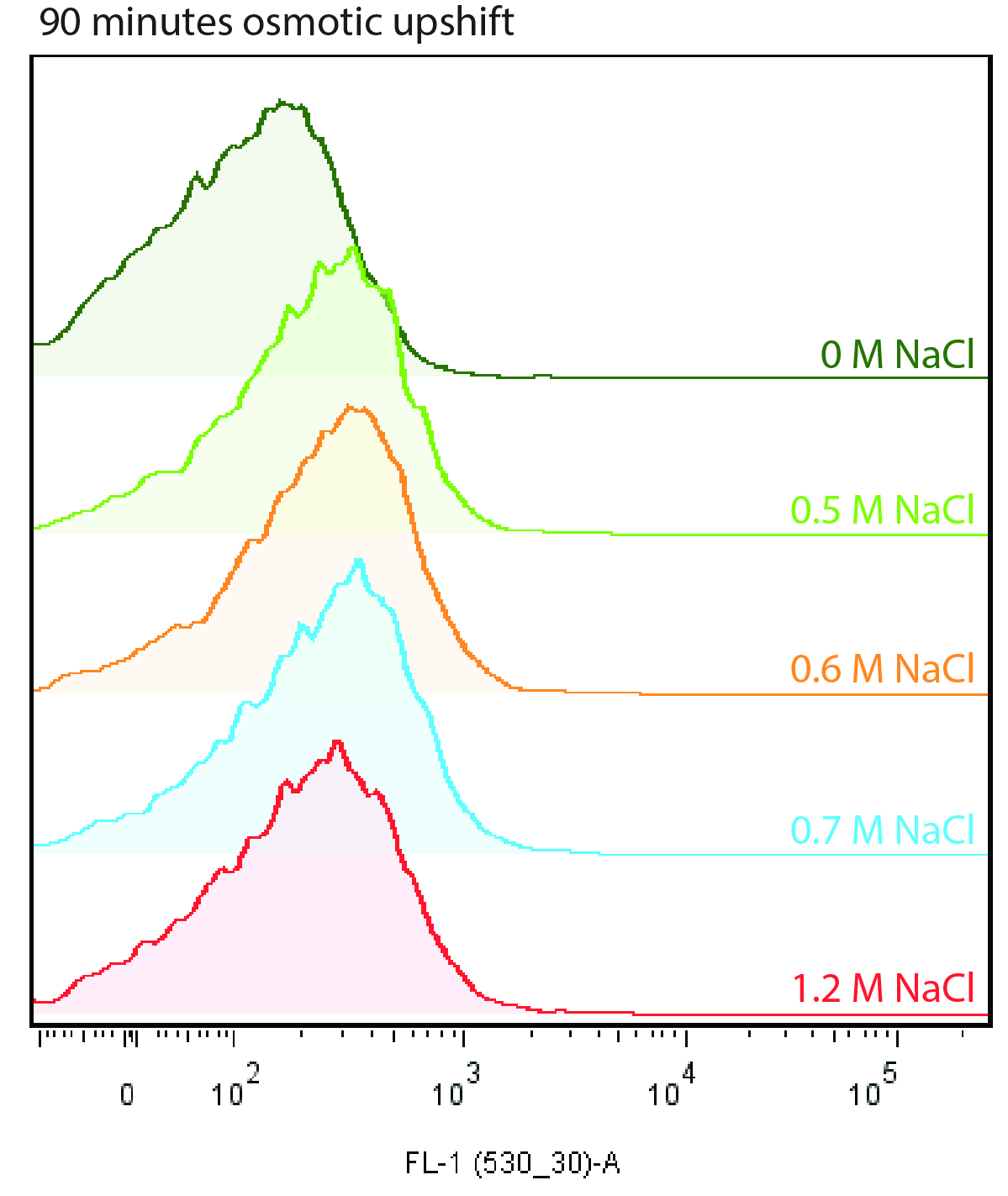 |
| --- |
| **Figure S1.** **Activation of the *proHJ* promoter in response to the osmotic upshift**  Fluorescence distribution of *B. subtilis* 168 P*proHJ*-*sfGFP (Sp)* strain in response to the different osmolarity of the environment after 90 minutes. The total sample size was 10.000 individual cells.   \| 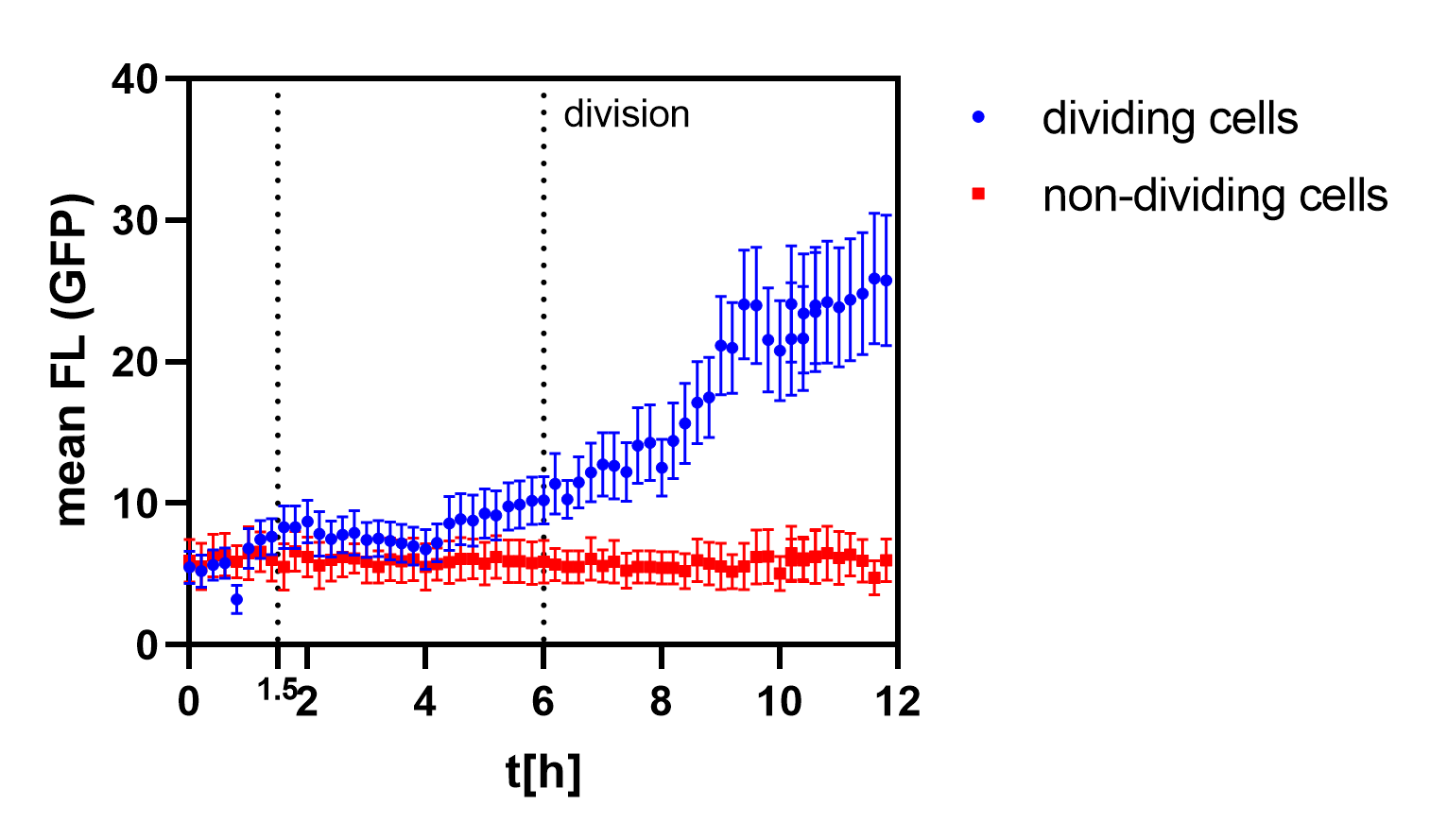 \| \| --- \| \| **Figure S2.** **Differences in the *proHJ* expression from dividing and non-dividing cells.**  The mean GFP fluorescent signal of dividing and non-dividing *B. subtilis* cells during 12 hours of exposure to the sustained osmotic upshift. 1.5 hours' time-point, indicated with a dotted line, shows the mean FL value for the early response to the osmotic upshift. The 6 hours' time-point marks the beginning of the GFP-positive cells division. The mean FL was measured with the MicrobeJ plugin for ImageJ, and the error bars show the SD of the measured signal dividing and non-dividing cells from a single frame. \| |
| 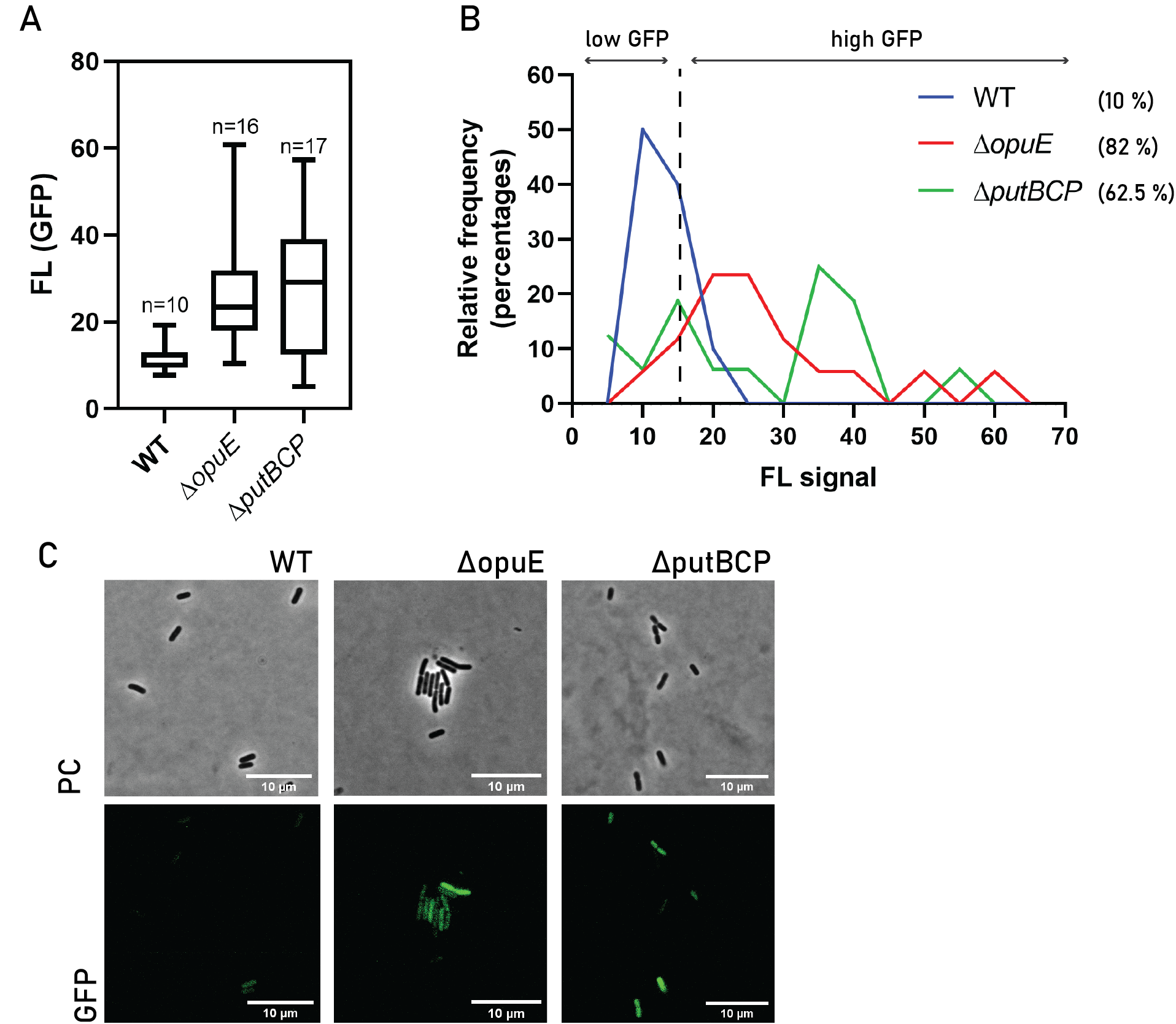 |
| **Figure S3. Activation of the *proHJ* promoter in osmotically stressed *B. subtilis* mutants.**  (A) Estimation of the average intensitiy of GFP fluorescent signal and (B) the distribution of the GFP fluorescent singal in studied populaions. The percentages brackets indicate the exact percentages of cells exhibiting high fluorescent singnal. (C) Micrographs of time-lapse microscopy showing adaptation of different mutants of *B. subtilis* to 0.6 M NaCl exposure at 90 minutes. The GFP fluorescent signal represents the activity of the *proHJ* promoter. The bar scale indicates 10 µm. |

**Genetic engineering of *B. subtilis* 168 variants**

*B. subtilis* 168 was prepared for transformation by growing a single colony, diluted in 2 ml of complete competence medium (1 X MC with 1 % tryptophan and 300 M MgSO_4_) (Table 4) for 17 hours. Subsequently, the culture was diluted to an OD600 of 0.05 in 20 ml competence medium and incubated for 5 hours until an OD600 of 0.7 was reached. Subsequently, ± 1 μg of plasmid DNA was added to 400 μl of culture, and cells were incubated for 2.5 hours to take up DNA. All plasmids used are listed in Table 2. Transformants were selected using LB agar plates with the appropriate antibiotic. Genomic DNA (gDNA) of *B. subtilis* was isolated according to the Promega protocol, except SET buffer (Table 4) that was used instead of BBA. The nucleotide sequence of the region of interest of the transformant gDNA was amplified by PCR and was verified by DNA sequence analysis using EZ-Seq (Macrogen, Amsterdam, The Netherlands).

All enzymes (for PCR, digestion, and ligation) were obtained from Thermo Fisher Scientific (Bleiswijk, The Netherlands). Polymerase Chain Reactions were performed with the use of Phusion High-Fidelity DNA polymerase and accompanying HF buffer according to New England Biolabs Protocol. All primers used are listed in Table 3. Enzymatic digestion was performed using FastDigest enzymes, and ligation of DNA fragments was done with T4 DNA (Thermo Fisher), according to the producer's protocols.

| **Table 1. Strains used in this study** | | | | |
| --- | --- | --- | --- | --- |
| **Strain** | **Species** | **Genotype** | **Ab^r^** | **Reference** |
| *B. subtilis* 168 P*proHJ*-*sfGFP (Sp)* | *B. subtilis* | tryptophan auxotroph *trpC2* | cm | This work |
| *B. subtilis* 168 | *B. subtilis* | tryptophan auxotroph *trpC2* | spec | BGSC |
| *E. coli* MC1061 | *E. coli* | F^–^ *araD*139, Δ (*ara*-*leu*)7696, *galE*15, *galK*16, Δ (*lac*)X74, *hsdR*2 (rK^–^mK^+^), *mcrA, mcrB*1, *rpsL* | - |  |
| **Table 2. Plasmids used in this study** | | | | |
| **Plasmid** | | **Genotype** | **Ab^r^** | **Reference** |
| pSG1151-P­*proHJ*-*gfpmut1* | | *bla, cat,* P­*proHJ*-*gfpmut1* | amp, cm | (Weme, 2015) |
| pSG1151-P*­proHJ*-*sfGFP (Sp)* | | *bla, cat,* P*­proHJ*-*sfGFP (Sp)* | amp, cm | This work |
| pKB01-*sfGFP (Sp)* | | *bla, tet, bga*A, P_Zn_-*sfgfp (Sp)* | amp, tet | (Overkamp et al., 2013) |
| **Table 3. Oligonucleotides used in this study** | | | | |
| Name | Sequence 5’ 🡪 3’ | | | Reference |
| proHJ_FW_SalI | CGATGGTCGACAAACAGCGGGATTATGGTCAAC | | | (Weme, 2015) |
| proHJ_REV_EcoRI | TCGGCGAATTCTTCCGCCATAGATCCTGCTC | | | (Weme, 2015) |
| sfGFPsp_for | GTCGAATTCATGTCAAAAGGAGAAGAACTTTTT | | | This work |
| sfGFPsp_rev | GGGACTAGTGCTCATTATTATTTATAAAGTTCG | | | This work |
| **Table 4. Media and buffers** | | | | |
| 10X MC media | 0.615 M K_2_HPO_4_ · 3H_2_O; 0.385 M KH_2_PO_4_;  20% glucose; 30 mM Tri-Na-citrate;  2% potassium glutamate;  1% casein hydrolysate;  0.02% Ammonium ferric citrate; | | | |
| SET buffer | 75 mM NaCl;  25 mM EDTA-Na_2_ · 2H_2_O; 20 mM Tris-HCl;  pH 7.5 | | | |
